# Cognitive Control Facilitates Attentional Disengagement during Second Language Comprehension

**DOI:** 10.3390/brainsci9050095

**Published:** 2019-04-27

**Authors:** Christian A. Navarro-Torres, Dalia L. Garcia, Vrinda Chidambaram, Judith F. Kroll

**Affiliations:** 1 Department of Psychology, University of California, Riverside, 900 University Avenue, Riverside, CA 92521, USA; dalia.garcia@ucr.edu (D.L.G.); jfkroll@gmail.com (J.F.K.); 2 Department of Comparative Literature and Languages, University of California, Riverside, 2401 HMNSS Building, Riverside, CA 92521, USA; vrinda@ucr.edu

**Keywords:** bilingualism, second language processing, sentence comprehension, cognitive control, attention, eye-tracking

## Abstract

Bilinguals learn to resolve conflict between their two languages and that skill has been hypothesized to create long-term adaptive changes in cognitive functioning. Yet, little is known about how bilinguals recruit cognitive control to enable efficient use of one of their languages, especially in the less skilled and more effortful second language (L2). Here we examined how real-time cognitive control engagement influences L2 sentence comprehension (i.e., conflict adaptation). We tested a group of English monolinguals and a group of L2 English speakers using a recently-developed cross-task adaptation paradigm. Stroop sequences were pseudo-randomly interleaved with a visual-world paradigm in which participants were asked to carry out spoken instructions that were either syntactically ambiguous or unambiguous. Consistent with previous research, eye-movement results showed that Stroop-related conflict improved the ability to engage correct-goal interpretations, and disengage incorrect-goal interpretations, during ambiguous instructions. Such cognitive-to-language modulations were similar in both groups, but only in the engagement piece. In the disengagement portion, the modulation emerged earlier in bilinguals than in monolinguals, suggesting group differences in attentional disengagement following cognitive control recruitment. Additionally, incorrect-goal eye-movements were modulated by individual differences in working memory, although differently for each group, suggesting an involvement of both language-specific and domain-general resources.

## 1. Introduction

Cognitive control is an essential feature of human cognition that enables appropriate goal-directed behavior through the regulation of basic thoughts and actions [1,2]. In recent years, the study of cognitive control has become central to our understanding of language processing [3,4,5,6]. For example, several empirical studies suggest an involvement of conflict resolution processes during lexical access [7,8,9], sentence production [10], and sentence comprehension [11,12,13,14]. Likewise, a number of lesion studies have established a link between cortical regions considered to be responsible for resolving interference and language abilities [15,16,17,18]. The engagement of such cortical regions has been observed across syntactic and non-syntactic tasks [6,19,20,21], suggesting that at least some of the control processes involved in language are domain-general in nature.

More recently, there has been an attempt to identify the conditions under which cognitive control is recruited online to facilitate language processing [22,23,24,25,26,27], with the evidence indicating that linguistic features differ in the demands they make on cognitive resources. Of interest to the present study are cases involving syntactic ambiguity during sentence comprehension and its relation to cognitive control. As individuals parse a sentence, meanings are rapidly assigned to words and phrases as the utterance unfolds in order to generate predictions about sentence-level meaning [28,29]. However, early interpretations can sometimes lead to erroneous predictions [30], forcing the comprehender to quickly revise and reinterpret the sentence. For example, in a title taken from a CNET online news article, “Google’s computer might betters translation tool”, the word “might” can act either as an auxiliary verb or as a noun. Since the former is more frequent than the latter by a ratio of about 134:1, people are more likely to initially assign the auxiliary verb interpretation, which is incompatible with the remainder of the sentence. Eventually “betters translation tool” signals a revision process to treat “might” as a noun, which can result in momentary unwanted interference stemming from the two incompatible interpretations. Under these conditions, cognitive control is hypothesized to facilitate the revision process, as well as the selection of the correct interpretation [31].

Perhaps one of the best sources of evidence for online engagement of cognitive control during the comprehension of syntactically ambiguous sentences comes from a study by Hsu and Novick [23]. Unlike most previous research, which relied on correlational analyses between measures of cognitive control and language performance, Hsu and Novick used a cross-task adaptation paradigm to test for a causal relation between the two domains. In this version of the paradigm, a button-press Stroop task was interleaved with visual-world sequences in which participants carried out spoken instructions by dragging and dropping objects around a visual scene while their eye-movements were recorded. Spoken instructions contained sentences that are known to induce momentary ambiguity, such as in the following example:

(1) Put the frog on the napkin onto the box.

Previous research has shown that in examples such as (1), adult listeners initially parse “on the napkin” as a destination [32,33]. However, hearing “onto the box” immediately signals a revision process in which “on the napkin” is treated as a modifying phrase, and not as a destination. Hsu and Novick argued that such revision process resulted in the momentary activation of two incompatible syntactic interpretations. They hypothesized that successful recovery from such interference stems from the recruitment of the same general-purpose control processes that would facilitate the processing of conflicting information in non-syntactic domains. If so, then participants’ comprehension of ambiguous instructions such as (1) should improve after experiencing Stroop-related conflict (i.e., when visual-world ambiguous sequences are preceded by incongruent Stroop sequences). In line with this hypothesis, eye-movement results revealed that incongruent, but not congruent, Stroop sequences facilitated participants’ ability to recover from ambiguous instructions. This was manifested as an increase in correct-goal fixations and as a decrease in incorrect-goal fixations.

The results from Hsu and Novick [23] illustrate the conditions through which the coordination of cognitive control becomes a critical resource for overcoming linguistic adversity; during sentence comprehension, the co-activation of multiple linguistic (e.g., semantic, lexical, phonological, and syntactic) features can lead to unwanted interference that must be resolved quickly through conflict resolution procedures in order to accomplish the task at hand. This hypothesis is also supported by previous research indicating that memory training improves online reading comprehension of syntactically ambiguous sentences, but only when the training engages conflict resolution processes [24,25]. Similar to Hsu and Novick [23], a more recent visual-world study by Thothathiri, Asaro, Hsu, and Novick [27] found that Stroop-related conflict facilitated the processing of ambiguous thematic role assignment during the comprehension of passive sentences. Taken together, these studies illustrate the dynamics of how language processing and cognitive control interact in the short-term. However, less is known about whether there are long-term experiences, such as lifelong language experience, that can influence the manifestation of such short-term effects.

One way to understand how language experience modulates the short-term dynamics between language processing and cognitive control is by examining bilinguals, given that actively learning and using a second language (L2) has long-term consequences for the language system [34]. Studies have shown that when bilinguals are speaking or comprehending, both languages are momentarily activated in parallel, even when the intention or requirement is to use only one [35,36,37]. One outcome of cross-language activation is that linguistic features of the language not in use can directly influence online performance in the other target language, even at high levels of proficiency [38,39]. For example, when linguistic (i.e., orthographic and/or semantic) features converge across the two languages, cross-language activation can result in online facilitation [40,41,42,43], although when features are in conflict with one another, such as in the case of homographs (i.e., words that overlap cross-linguistically in orthography but differ in meaning, e.g., the word pan means bread in Spanish) or partially overlapping syntactic structures, cross-language activation can produce momentary online interference [44,45,46,47].

An issue of interest to the present study is how bilinguals manage to regulate and suppress cross-language interference in order to successfully process ambiguity in one language, especially in the less skilled and more effortful L2. Prior research suggests that, when processing syntactic ambiguity, L2 speakers have greater difficulties revising misinterpretations [48] and exhibit different (or greater variability in) attachment preferences from native (L1) speakers [49,50,51]. Although general differences between L1 and L2 processing have been previously explained in terms of processing constraints in the L2 [52], more recent evidence suggests that both languages engage the same neural and cognitive processes [53,54,55] and that these differences reflect variability in proficiency [56], speed of lexical access [57], and cognitive control ability [14]. In this sense, L2 processing may be more susceptible to irrelevant (within-language and cross-language) interference [58], given that both languages compete for cognitive resources. Given these constraints, highly proficient L2 comprehension may provide a unique window to understand different aspects of cognitive control recruitment during the processing of syntactic ambiguity, perhaps in a way that may not be easily observed in monolinguals.

For the reasons outlined above, mastery of L2 processing seems to largely depend on bilinguals’ ability to regulate language-related conflict [59,60]. The control mechanisms that mediate such conflict are hypothesized to be domain-general in nature, a claim that is largely supported by neuroimaging research [61,62,63,64]. Such interplay is further hypothesized to create long-term consequences for cognitive functioning [65]. Recently, there has been an interest in understanding how bilingual experience influences the control processes associated with conflict monitoring and attentional disengagement. For example, recent studies have shown that bilinguals develop high efficiency in disengaging misleading or irrelevant information after experiencing conflict in both linguistic [66,67] and non-linguistic tasks [68,69,70]. Bilinguals are also more likely to perform differently from monolinguals under conditions of high conflict that require careful monitoring [71,72,73]. However, this research has focused on examining the long-range consequences on cognitive functioning, where it is assumed that cognitive control processes gradually adapt to the demands imposed by the language practices that bilinguals adopt [64]. What is less understood is how cognitive control procedures can be utilized by bilinguals in real time to support language processing, which may be critical for better understanding the long-term adaptation associated with becoming bilingual.

### Present Study

In the present study, we examine whether cognitive control processes modulate how bilinguals experience syntactic ambiguity in their L2, compared to native speakers of that language. We adopt the cross-task adaptation paradigm used in Hsu and Novick [23], which utilizes a combination of a behavioral/cognitive (i.e., Stroop) task and a visual-world paradigm [28,74], both of which are pseudo-randomly interleaved within the same experimental block. A key feature of the visual world paradigm is that it allows us to examine the way in which an unfolding sentence can guide visual attention, which is effortful in and of itself. We test the hypothesis that conflict experienced in a non-syntactic task will trigger cognitive control procedures that will facilitate subsequent performance in a syntactic task. But we additionally explore the idea that bilinguals may engage in this process in a different manner, as a direct result of having to mediate unique demands that are imposed on the L2 system.

Similar to the idea of conflict adaptation, bilinguals may be able to disengage attention more efficiently immediately following conflict in ways that are not typically observed in monolinguals [68,69,72]. Such an ability may stem from a complex coordination of multiple executive functions, which may be difficult to characterize depending on the task. However, the visual world paradigm is an ideal tool to examine and dissociate engagement from disengagement processes. For example, the sentence ‘put the frog on the napkin onto the box’ could be accompanied by a visual display in which either one or two frogs are presented. In the former case, adult listeners’ ability to engage attention in the correct destination object (a box) is typically delayed because they have a difficult time disengaging their attention from the incorrect destination object (i.e., an empty napkin) [32,75]. For monolinguals, the ability to navigate through both sources of information seems to be equally facilitated by cognitive control [23,27]. However, for bilinguals, it may be possible to observe a dissociation between the two processes when conflict resolution resources are readily available. This may be especially true for bilinguals who speak English as the L2, since, as previously mentioned, L2 sentence processing is likely to be more susceptible to interference [56].

In the present study, we focus on a group of proficient bilinguals, all of whom were born and raised in non-English-speaking environments but began acquiring English after their L1. These individuals eventually became immersed in English (i.e., Edinburgh, Scotland) as part of their higher education, although they maintained L1 dominance. Therefore, we hypothesized that, for these bilinguals, online comprehension in English would be more effortful relative to monolinguals (likely manifesting greater fixation costs, especially when parsing ambiguous sentences). However, we further hypothesized that these added costs would create a greater need for cognitive control recruitment, which might yield group differences in the timing and/or magnitude of the recovery from ambiguity.

## 2. Materials and Methods

### 2.1. Participants

Participants included a total of 26 English monolinguals and 24 bilinguals who acquired English as the L2. Monolinguals were fluent in English only, had not enrolled in any second-language courses during college, and had no more than minimal exposure to an L2 prior to college. Bilinguals were all born and raised in a non-English speaking country and reported using English as the L2 and another language as the L1. These languages included Arabic (1), Cantonese (2), Cypriot Greek (1), Dutch (1), German (3), Greek (1), Italian (7), Mandarin (1), Norwegian (2), Romanian (1), Russian (1), and Spanish (3). Monolinguals were recruited either at the Pennsylvania State University or at the University of California, Riverside, and received either course credit or $10/hour. Bilinguals were recruited at the University of Edinburgh and received an equivalent of $10/hour in pounds. All participants gave informed consent, and the procedures had the approval of the Institutional Review Board of the Pennsylvania State University and of University of California, Riverside.

Table 1 provides the characteristics of participants for both groups. Participants completed a category fluency task and the operation span (O-span) task [76] to assess verbal abilities and working memory abilities, respectively. In the category fluency, participants were asked to generate as many exemplars as possible that belong to a semantic category within a 30-second time limit. Monolinguals were presented with four out of eight possible categories (counter-balanced across participants), and bilinguals were presented with four unique categories for the L1 and for the L2 in separate blocks. In the O-span task, participants attempted to solve math equations while simultaneously memorizing words in English that would have to be recalled later in the task. They also completed a language history questionnaire to assess self-rated levels of language proficiency, among other characteristics.

Bilinguals were slightly older than the monolinguals (*t* (47) = −2.98, *p* = 0.005), although both groups consisted of university students. Bilinguals reported acquiring English around age 6 and also reported becoming immersed in an English environment in recent years as part of their education at the University of Edinburgh. Self-rated L1 exposure (*t* (21) = −4.99, *p* < 0.001) and L1 use (*t* (21) = −3.83, *p* = 0.001) were relatively low compared to the L2, suggesting restricted access to the L1 likely due to L2 immersion. Despite this, bilinguals self-rated themselves as being more dominant in their L1 (*t* (21) = 5.60, *p* < 0.001), and displayed marginally higher verbal fluency in their L1 than in their L2 (*t* (20) = 1.86, *p* = 0.077). Compared to monolinguals, bilinguals had lower English fluency (*t* (45) = 5.31, *p* < 0.001), and lower working memory ability (*t* (47) = 5.46, *p* < 0.001), although both groups’ scores fell within typical ranges. Both groups, however, had similar basic processing speed abilities, as indexed by the response times for solving math equations in the O-span (*t* (48) = −0.381, *p* = 0.705). The differences in working memory recall, however, are expected considering that working memory has been shown to differ in the L1 vs. the L2 [77].

### 2.2. Materials

The materials used for the cross-task adaptation paradigm in our study were taken from Hsu and Novick [23]. No modifications were made to the materials. In this version of the paradigm, a Stroop task is interleaved with a sentence comprehension task that involves syntactic ambiguity. During the sentence comprehension portion, participants listened to spoken instructions in English while viewing a series of objects on the screen, some of which were mentioned in the instructions. The goal of the participants was to carry out the spoken instructions by clicking and dragging objects around the screen accordingly. Critical trials involved instructions whose meaning was ambiguous or unambiguous. Each critical trial was preceded by Stroop sequences in which the ink color was either congruent or incongruent.

In the Stroop portion, participants used a three-button mouse to indicate the ink color of the word as quickly as possible. The response set consisted of blue, green, and yellow. Participants were randomly assigned to four versions that contained different button-color mappings. There was a total of 120 Stroop sequences, half of which were congruent. For the 60 incongruent sequences, color names never matched the colors used in the response set (i.e., blue, green, and yellow were never used as color names).

**Figure 1:**
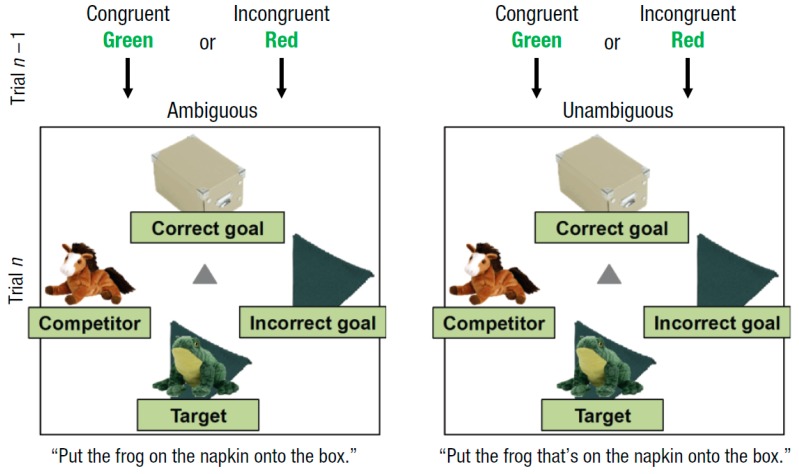
Illustration of the experimental design depicting a Stroop-to-sentence sequence. Figure taken from Hsu and Novick [23].

In the sentence comprehension portion, the instructions were prerecorded by a female speaker, which contained single sentences such as “put the frog on the napkin onto the box” (ambiguous) or “put the frog that’s on the napkin onto the box” (unambiguous). In these two examples (see Figure 1), the visual scene contained a frog on a napkin (target), an empty napkin (incorrect goal), a box (correct goal), and a horse (competitor). This setup created a context that favored a goal analysis (i.e., treating “on the napkin” as the correct goal) instead of a modifier analysis (i.e., treating “on the napkin” as a modifying clause of “frog”). The omission of the complementizer “that’s” would therefore create momentary ambiguity. In this scenario, hearing “onto the box” would force the comprehender to revise the initial interpretation of the sentence.

There was a total of 24 ambiguous and 24 unambiguous sentences. These sentences were pseudo-randomly interleaved with Stroop sequences. Half of each sentence type was preceded by congruent Stroop sequences, and the other half was preceded by incongruent sequences. Therefore, critical trials were manipulated for sentence type (whether the instruction was ambiguous or unambiguous) and preceding Stroop trial type (whether the instruction was preceded by a congruent or incongruent Stroop sequence), meaning that each of these experimental conditions contained 12 trials total. There were 48 additional filler sentences that would minimize the saliency of the critical manipulation. These filler sentences always contained “put” instructions such as “put the walrus on/onto/under the desk” or “put the monkeys next to each other”. This was done to ensure that participants did not automatically treat “on the napkin” as a reduced relative clause, since the locative prepositional phrases in filler trials would contain the correct goal. Like critical trials, filler sentences were preceded by Stroop sequences, meaning that participants could not anticipate the type of sentence based on Stroop trial type. Out of the 120 Stroop sequences, 72 were used to create Stroop-to-Stroop sequences (ranging from 2 to 4 consecutive Stroop sequences) to make the alternation between the two tasks less predictable. Together with the fillers, the interleaved design created several Stroop-to-Stroop (28%), Stroop-to-Sentence (27%), Sentence-to-Stroop (28%), and Sentence-to-Sentence (17%) pairings.

Some pictures were repeated across conditions, but each item combination (e.g., frog-napkin-box or mug-blanket-doormat) occurred only once. Goal-depicting objects (e.g., napkin, box) were counterbalanced such that they could appear in the sentence as either the target (first noun phrase), incorrect goal (second noun phrase), or correct goal (third noun phrase). Object location was counterbalanced within and across conditions. Two lists were also created in which sentence ambiguity was counterbalanced within items (i.e., an object that appeared in the ambiguous condition in one list would appear in the unambiguous condition on the other list).

### 2.3. Procedure

Participants were tested in a sound-attenuated room and were seated in front of a computer monitor. Stimuli for the main task were presented using Experiment Builder software (Version 2.1.140, SR Research, Kanata, ON, Canada). Eye-movements were recorded with an EyeLink 1000 eye-tracker at the University of Edinburgh and an EyeLink 1000 plus at the Pennsylvania State University and at the University of California, Riverside (SR Research, Ottawa, Canada; temporal resolution: 1000 Hz; spatial resolution: ≤ 1.5°). Stimuli for the secondary tasks were presented in a separated computer monitor that was connected to a button box and a digital recorder.

Participants first completed the main interleaved task, followed by category fluency, O-span, and the language history questionnaire. Before starting the main task, participants were given a practice block of 144 Stroop trials to familiarize themselves with the color-button mapping that was assigned to them. Immediately after, they were given four practice trials containing visual world sequences. In the main task, each trial began with the mouse cursor appearing as a fixation point in the middle of the screen for 500 ms. For Stroop sequences, the cursor was replaced by a stimulus item, which remained on the screen for 1000 ms or until the participant clicked on one of the mouse buttons. For the sentence comprehension sequence, the cursor was replaced by visual objects on the screen, all of which could be moved as soon as they appeared. After a 300 ms delay, participants began hearing the commands for that trial. During the Stroop portion, participants were encouraged to press the mouse button corresponding to the ink color of the word as quickly as possible. During the sentence comprehension portion, participants were told that there would be a limited time to follow and carry out the spoken instructions using the mouse to drag objects around the screen, and were encouraged to do so as accurately as possible.

### 2.4. Analysis

Accuracy and response time data were collected for Stroop trials. For the sentence comprehension task, eye-movements were recorded. Following Hsu and Novick [23], each quadrant of the screen was labeled as an interest area. Proportion of fixations were subsequently obtained by creating sample reports using the EyeLink Data Viewer Tool (Version 3.1.97; SR Research, Ottawa, Canada). For the purposes of the paper, we focus on analyzing eye-movements given that our main interest was to examine how cognitive control affects sentence comprehension in real time. We excluded trials with more than 33% loss in the eye-tracking data (4.9% of the data set).

We calculated the proportion of fixations to the correct and incorrect goal across seven time points. This included an action period, the final fixation point, corresponding to the offset of the sentence when participants were able to complete the mouse movements (e.g., 1: Put the, 2: frog (that’s), 3: on the, 4: napkin, 5: onto the, 6: box; 7: action period). We followed a procedure similar to Hsu & Novick [23], where correct and incorrect goal fixations were analyzed separately. The analysis consisted of aggregating fixations across the last four time points (i.e., at the onset of “napkin” all the way through the action period). Hsu & Novick [23] conducted two separate analyses, one including correct and incorrect action responses, and another only including correct action responses. In the present study, all analyses included both correct and incorrect action responses. Fixations were analyzed with mixed effects models using the lme4 software package [78] in the R programming environment [79]. The main analyses included contrast coded fixed effects of sentence type (ambiguous = −0.5, unambiguous = 0.5), preceding Stroop trial type (congruent = −0.5, incongruent = 0.5), group (monolinguals = 0.5, bilinguals = −0.5), and their interaction. In a second set of analyses, we added a fixed effect of experimental half (first = 0.5, second = −0.5) to examine the modulation of the effects of interest across time, an analysis that proved to be critical in Hsu and Novick [23]. All models included crossed random effects for participants and items. We attempted to fit random effects using a maximal procedure [80]. However, due to convergence failures and problems with singularity, some random slopes had to be removed to attain convergence. We report the full results of each analysis, including fixed effects and random effects estimates, in the Supplementary Materials.

Fixations were transformed using an empirical logit function to account for the bounded nature of proportions. Significance of the coefficients was determined using the Satterthwaite approximation with the lmerTest package, version 3.0-1 [81]. Significant interactions and follow-up comparisons were examined by refitting the model with a dummy coded categorical factor to examine simple effects at each level of the categorical factor. Note that refitting the model simply re-estimates the parameters with a different reference point without affecting the goodness of fit or the type-1 error rate. Instead, it simply provides a different interpretation of the coefficients while keeping the variance constant [82].

## 3. Results

### 3.1. Fixations on the Correct and Incorrect Goal

**Figure 2:**
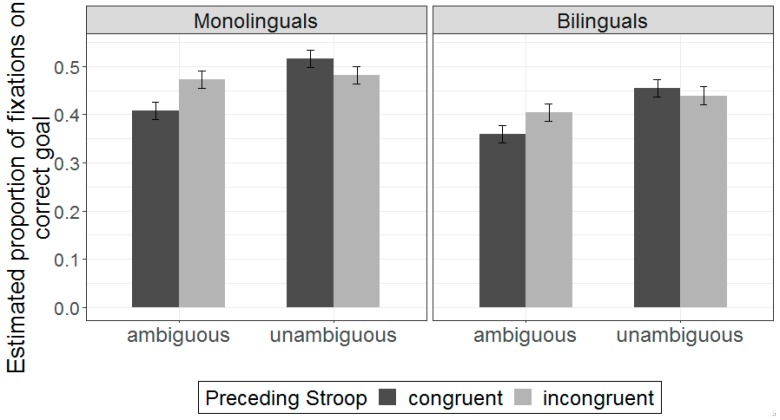
Estimated effects for correct-goal fixations for ambiguous sentences as a function of prior Stroop trial type. Higher values on the y-axis indicate more looks to the correct goal object (e.g., a box. Fixations were averaged from the onset of the second noun phrase (e.g., napkin) through the action period. Error bars indicate standard error of the mean.

Table 2 shows the mean proportion of fixations on the correct and incorrect goal for each group across conditions. Figure 2 shows estimated effects for the correct-goal analysis across conditions (see also Figures S1 and S2 in the Supplementary Materials). The full model description for the correct goal analysis is shown on Table S1. There was a main effect of ambiguity (β = 0.09, SE = 0.02, *t* = 6.05, *p* < 0.001), indicating that the looks to the correct goal were overall lower for ambiguous sentences compared to unambiguous sentences (Figure S1). There was also a main effect of group (β = 0.09, SE = 0.02, *t* = 6.05, *p* < 0.001), suggesting that bilinguals had overall reduced correct goal fixations relative to monolinguals. There was also a significant interaction between ambiguity and preceding Stroop trial type (β = −0.10, SE = 0.03, *t* = −3.02, *p* = 0.004). Simple effects analyses revealed that correct goal fixations increased for ambiguous sentences when preceded by incongruent Stroop sequences relative to congruent Stroop sequences (β = 0.26, SE = 0.09, *t* = 2.94, *p* = 0.004), but no such effect was observed for unambiguous sentences (β = −0.11, SE = 0.09, *t* = −1.32, *p* = 0.190). Additionally, the ambiguity effect was larger for trials preceded by congruent Stroop sequences (β = 0.14, SE = 0.02, *t* = 5.86, *p* < 0.001) compared to trials preceded by incongruent Stroop sequences (β = 0.04, SE = 0.02, *t* = 2.25, *p* = 0.031). Confidence intervals around the estimates also indicated that the ambiguity effect was different across the two preceding Stroop conditions (congruent Stroop: 95% CI [0.09, 0.19]; incongruent Stroop: 95% CI [0.01,0.08]), suggesting that the facilitating effect of Stroop-related conflict minimized fixation differences between ambiguous and unambiguous sentences. The main effect of preceding Stroop trial was not significant, suggesting that Stroop performance impacted fixations patterns only when following incongruent-to-ambiguous sequences. Group did not interact with any of the other variables in the model, suggesting that this pattern of results was relatively similar across the two groups.

**Figure 3:**
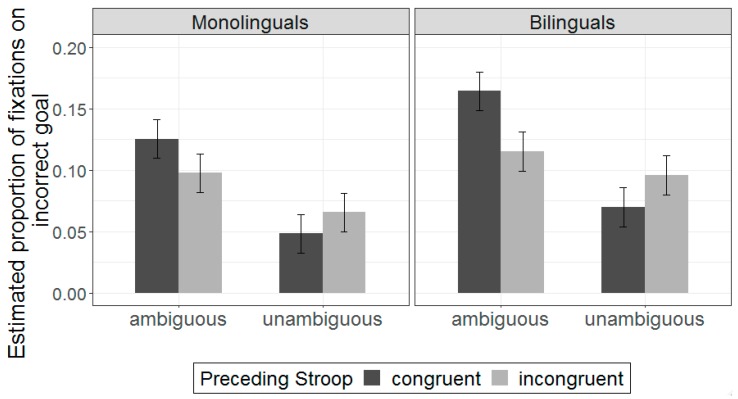
Estimated effects for incorrect-goal fixations for ambiguous sentences as a function of prior Stroop trial type. Higher values on the y-axis indicate more looks to the incorrect goal object (e.g., an empty napkin). Fixations were averaged from the onset of the second noun phrase through the action period. Error bars indicate standard error of the mean.

Next, we examine incorrect goal performance, which is shown on Figure 3 (see also Figures S3 and S4). The full model description is shown on Table S2. In general, the results were similar to those observed in the correct goal analysis, although group differences emerged. There was a main effect of ambiguity (β = −0.13, SE = 0.02, *t* = −5.61, *p* < 0.001), indicating that hearing ambiguous instructions resulted in increased looks to the incorrect goal relative to unambiguous instructions (Figure S3). The main effect of group was again significant (β = −0.07, SE = 0.02, *t* = −2.95, *p* = 0.005), suggesting that, overall, bilinguals spent more time fixating on the incorrect goal than monolinguals. The interaction between sentence type and preceding Stroop trial was also significant (β = 0.09, SE = 0.04, *t* = 2.08, *p* = 0.046), although a marginally significant three-way interaction between sentence type, preceding Stroop trial, and group also emerged (β = −0.08, SE = 0.04, *t* = −1.96, *p* = 0.050).

Follow-up analyses revealed that the interaction between sentence type and preceding Stroop trial type was only significant for bilinguals (β = 0.13, SE = 0.05, *t* = 2.75, *p* = 0.008) and not monolinguals (β = 0.05, SE = 0.05, *t* = 1.02, *p* = 0.312). Like in the correct goal analysis, for bilinguals, the effect of preceding Stroop trial was only significant for ambiguous sentences (β = −0.10, SE = 0.04, *t* = −2.51, *p* = 0.015), such that incorrect goal fixations decreased when ambiguous instructions were preceded by incongruent Stroop sequences. No such effect was observed for unambiguous sentences (β = 0.02, SE = 0.03, *t* = 0.92, *p* = 0.359).

The three-way interaction revealed a second group difference. For bilinguals, when examining trials preceded by congruent Stroop sequences, the expected effect of ambiguity was significant (β = 0.19, SE = 0.04, *t* = 5.08, *p* < 0.001), but the difference between ambiguous and unambiguous sentences was reduced for trials preceded by incongruent Stroop sequences (β = −0.07, SE = 0.03, *t* = −1.92, *p* = 0.060). For monolinguals, however, differences between ambiguous and unambiguous sentences remained regardless of the preceding Stroop sequence (congruent Stroop: β = −0.15, SE = 0.04, *t* = −3.94, *p* < 0.001; incongruent Stroop: β = −0.10, SE = 0.03, *t* = −3.00, *p* = 0.004). Additionally, although the effect of group was not significant for congruent-unambiguous sequences (β = 0.06, SE = 0.03, *t* = 1.82, *p* = 0.071), there was a significant group difference for incongruent-unambiguous sequences (β = 0.08, SE = 0.03, *t* = 3.08, *p* = 0.003). This pattern of results suggests that, for unambiguous sentences, Stroop-related conflict may have inadvertently increased bilinguals’ looks to the incorrect goal.

**Figure 4:**
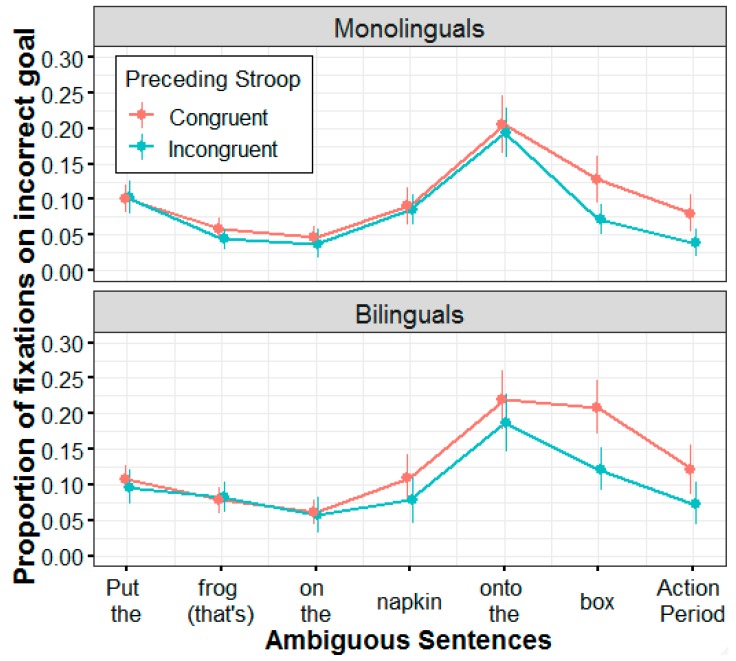
Mean proportion of fixations on the incorrect goal over time for ambiguous sentences as a function of preceding Stroop trial type. Higher values on the *y*-axis indicate more looks to the incorrect goal. Colored error bars indicate 95% confidence intervals.

**Figure 5:**
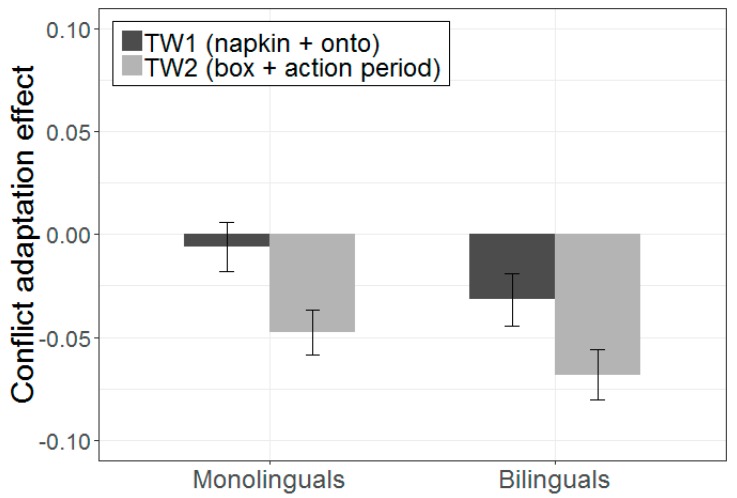
Effect of conflict adaptation (calculated as the difference between mean proportion of incongruent-ambiguous fixations and mean proportion of congruent-ambiguous fixations) for the incorrect goal during an early (TW1) and late (TW2) time window. More negative values on the *y*-axis indicate greater difference between incongruent-ambiguous and congruent-ambiguous sequences and, therefore, greater facilitation from incongruent Stroop sequences. Error bars indicate standard error of the mean.

The incorrect-goal results obtained thus far suggest important group differences in the effect of recovery from ambiguity following Stroop-related conflict. It is possible that this is due to differences in the magnitude of recovery from ambiguity, although group differences can also arise with respect to when the recovery process began. To further address this issue, we examined the effect of conflict adaptation (by subtracting incongruent-to-ambiguous fixations from congruent-to-ambiguous fixations) in two time windows: an early time window (TW1) that included fixations from the second noun phrase (e.g., napkin) and the disambiguating region (e.g., onto the), and a later time window (TW2) that included fixations from the third noun phrase (e.g., box) and the action period (see Figure 4 and Figure 5).

A repeated measures ANOVA (with time window as a within-subjects factor, and group as a between-subjects factor) revealed a main effect of time window (*F* (1, 48) = 11.26, *p* = 0.002, η^2^ = 0.19), indicating that the overall magnitude of recovery was weaker at TW1 (M = −0.02) and increased at TW2 (M = −0.06). A main effect of group indicated that the overall magnitude of recovery was greater for bilinguals (M = −0.05) than for monolinguals (M = −0.02), although this effect was only marginally significant (*F* (1, 48) = 3.42, *p* = 0.071, η^2^ = 0.07). The interaction between time window and group was not significant (*F* (1, 48) = 0.08, *p* = 0.776, η^2^ = 0.00). However, as Figure 4 and Figure 5 suggest, recovery started earlier for bilinguals than for monolinguals. One sample *t*-tests revealed that, for bilinguals, the effect of conflict adaptation was significantly different from zero at both TW1 (*t* (23) = −2.41, *p* = 0.024) and TW2 (*t* (23) = −5.63, *p* < 0.001), but for monolinguals this effect was significant at TW2 (*t* (25) = −4.43, *p* < 0.001) but not at TW1 (*t* (25) = −0.431, *p* = 0.670). Taken together, these results suggest that the group differences are more likely to reflect differences in the timing of cognitive control engagement, and not necessarily in the magnitude of the effect.

### 3.2. Conflict Adaptation during the First and Second Half of the Experiment

The results reported so far suggest that both bilinguals and monolinguals engaged cognitive control in a similar manner with respect to correct goal fixations, but differed in terms of how control was recruited when fixating on the incorrect goal. Which aspects of cognitive control can account for such discrepancies? One possibility is that reliance on task disengagement procedures maximized bilinguals’ ability to shift away from incorrect interpretations [54,64]. Another possibility is that bilinguals relied on conflict monitoring procedures to a greater extent than monolinguals [71,72,73]. This latter account is particularly interesting, since it would predict for bilinguals general sustained effects of conflict adaptation throughout the experiment. We attempted to dissociate these two alternatives by examining how Stroop performance and fixations are modulated by time (here defined as the first and second half of the experiment).

We first report an analysis of Stroop response time data in the first and second half of the experiment (Table 3). There was a significant Stroop effect in the first half of the experiment for both monolinguals (β = 0.03, SE = 0.01, *t* = 2.08, *p* = 0.038) and bilinguals (β = 0.04, SE = 0.01, *t* = 2.71, *p* = 0.007), indicating that incongruent trials resulted in slower latencies relative to congruent trials. However, the Stroop effect disappeared in the second half of the experiment for both groups (monolinguals: β = 0.02, SE = 0.01, *t* = 1.18, *p* = 0.236; bilinguals: β = −0.00, SE = 0.02, *t* = −0.22, *p* = 0.826). This is consistent with previous literature showing that Stroop costs dissipate over time due to practice effects [83].

**Figure 6:**
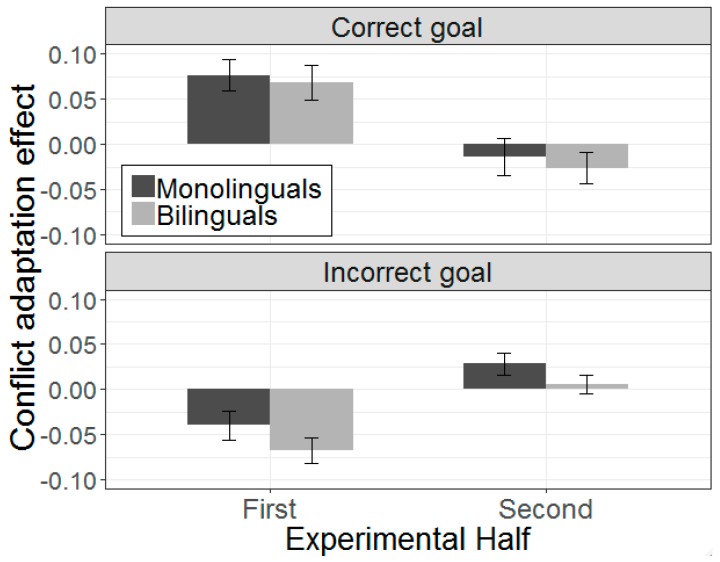
Effect of conflict adaptation on the correct (top) and incorrect (bottom) goal in monolinguals and bilinguals, split by the first and second half of the experiment. Greater values (i.e., more positive for the correct goal, and more negative for the incorrect goal) on the *y*-axis reflect greater difference between incongruent-ambiguous and congruent-ambiguous sequences and, therefore, greater facilitation from incongruent Stroop sequences. Error bars indicate standard error of the mean. Error bars indicate standard error of the mean.

In the correct-goal analysis (Table S3), the three-way interaction between preceding Stroop trial type, sentence type, and experimental half was significant (β = −0.23, SE = 0.09, *t* = −2.59, *p* = 0.010). During the first half of the experiment, the effect of preceding Stroop trial type was significant for ambiguous sentences (β = 0.13, SE = 0.04, *t* = 3.50, *p* < 0.001), reflecting conflict adaptation. But as Figure 6 suggests, this modulation disappeared in the second half of the experiment (β = −0.03, SE = 0.04, *t* = −0.81, *p* = 0.420). A similar pattern was observed in the incorrect-goal analysis (Table S4 and Figure 6). The three-way interaction between Stroop trial type, sentence type, and experimental half was again significant (β = −0.23, SE = 0.09, *t* = −2.59, *p* = 0.010), indicating an effect of preceding Stroop for ambiguous sentences in the first half (β = −2.24, SE = 0.05, *t* = −2.24, *p* = 0.026) but not in the second half (β = 0.04, SE = 0.05, *t* = 0.76, *p* = 0.448) of the experiment. Although the three-way interaction between Stroop trial type, sentence type, and group was again significant (β = −0.09, SE = 0.05, *t* = −2.07, *p* = 0.038), the four-way interaction including experimental half was not (β = −0.06, SE = 0.09, *t* = −0.71, *p* = 0.473), suggesting that the recovery effect following incongruent-ambiguous sequences was similar in both groups (although numerically smaller in monolinguals) once time is taken into account.

No reliable effects of preceding Stroop were observed for unambiguous sentences in either half of the experiment. This was true for both correct-goal (first half: β = −0.04, SE = 0.04, *t* = −1.22, *p* = 0.224; second half: β = −0.03, SE = 0.04, *t* = −0.94, *p* = 0.349) and incorrect-goal (first half: β = −0.06, SE = 0.04, *t* = −1.53, *p* = 0.133; second half: β = 0.01, SE = 0.05, *t* = 0.26, *p* = 0.793) fixations. Moreover, group did not interact with any of the other factors, suggesting that these modulations were similar for both groups. Consistent with Hsu and Novick [23], these results suggest that Stroop-induced conflict was responsible for modulating the ambiguity effect in both groups with respect to correct and incorrect-goal fixations. They also suggest that the discrepancies between monolinguals and bilinguals in the main analysis were due to differences in the recruitment of task disengagement processes, and not necessarily due to differences in conflict monitoring abilities.

### 3.3. Individual Differences in Working Memory

Although it is reasonable to assume that L1 and L2 comprehension make use of the same cognitive resources, the incorrect-goal results suggest that differences might arise in the way those resources are recruited. One way to further explore this idea is by examining whether (and how) working memory ability mediates online comprehension, especially since working memory is often found to modulate syntactic ambiguity in both L1 and L2 speakers [84,85]. To do this, we conducted exploratory mixed model analyses for correct and incorrect goal fixations, adding O-span recall scores as a predictor, and allowing it to interact with the fixed effects included in previous models.

**Figure 7:**
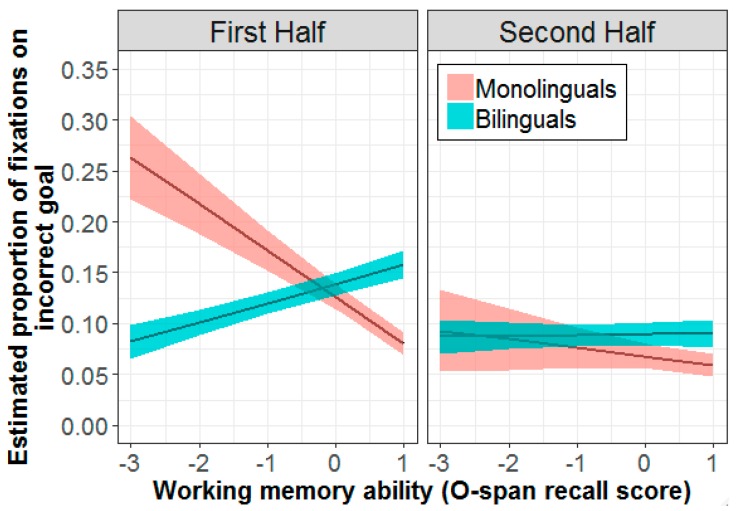
Estimated effect of working memory on incorrect-goal fixations in the first (left) and second (right) half of the experiment for bilinguals (*n* = 23) and monolinguals (*n* = 26). More positive values on the *x*-axis indicate higher O-span recall scores. Shaded areas indicate standard error of the mean.

Although no effects of working memory were found for correct-goal fixations, a strong pattern of association between O-span and incorrect-goal fixations emerged for both groups (Table S5). There was a significant interaction between group and O-span (β = −0.09, SE = 0.02, *t* = −3.60, *p* < 0.001), indicating that, for monolinguals, higher O-span recall scores were associated with a decrease in incorrect-goal fixations (β = −0.06, SE = 0.02, *t* = −2.88, *p* = 0.004). For bilinguals, however, higher O-span was associated with an increase in incorrect-goal fixations (β = 0.02, SE = 0.01, *t* = 2.28, *p* = 0.024). A three-way interaction between group, O-span, and experimental half (Figure 7) indicated that these associations emerged for the first half (monolinguals: β = −0.10, SE = 0.03, *t* = −3.88, *p* < 0.001; bilinguals: β = 0.04, SE = 0.01, *t* = 3.33, *p* < 0.001) but not for the second half (monolinguals: β = −0.02, SE = 0.03, *t* = −0.81, *p* = 0.421; bilinguals: β = 0.01, SE = 0.01, *t* = 0.38, *p* = 0.703) of the experiment. These patterns of associations indicate that working memory differentially impacted each group’s ability to disengage incorrect-goal fixations.

**Figure 8:**
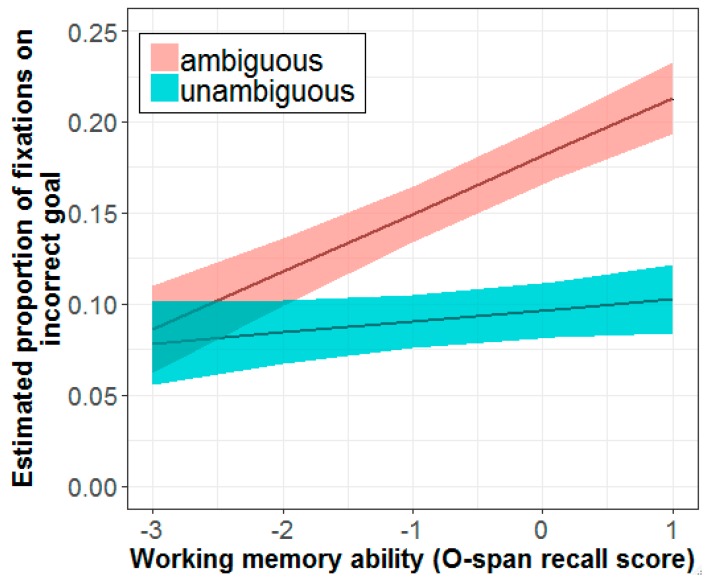
Estimated effect of working memory on incorrect-goal fixations for ambiguous sentences in bilinguals (*n* = 23). More positive values on the x-axis indicate higher O-span recall scores. Shaded areas indicate standard error of the mean.

Perhaps more interesting was the four-way interaction between sentence type, group, experimental half, and O-span (β = 0.16, SE = 0.07, *t* = 2.18, *p* = 0.030), which yielded a three-way interaction between sentence type, group, and O-span for the first experimental half (β = 0.16, SE = 0.07, *t* = 2.18, *p* = 0.030). As shown in Figure 8, for bilinguals, the effect of O-span in the first experimental half was significant for ambiguous sentences (β = 0.07, SE = 0.02, *t* = 3.69, *p* < 0.001) but not for unambiguous sentences (β = 0.02, SE = 0.02, *t* = 0.97, *p* = 0.333). The pattern of association for monolinguals did not differ across conditions, indicating that the effect of working memory on fixation patterns was more global, whereas for bilinguals the effect was primarily driven by ambiguous sentences.

## 4. Discussion

In an eye-tracking experiment examining cognitive control recruitment during sentence comprehension, we found that Stroop-induced conflict facilitated recovery from syntactic ambiguity. As a result, both monolinguals and bilinguals were better able to engage correct-goal interpretations as they revised their initial misinterpretations in real time. This advantage in recovery was observed only in the presence of a Stroop effect, which emerged during the first half of the experiment, but disappeared afterwards. These findings directly replicate the results from Hsu and Novick [23]. Similarly, Stroop-related conflict modulated the ability to disengage incorrect-goal interpretations during recovery from syntactic ambiguity, but this process began earlier for bilinguals. In fact, the effect was not statistically reliable for monolinguals in the initial analysis, which is at odds with the results reported by Hsu and Novick [23].

What factors account for such discrepancies? One possibility is that, at least in the context of this paradigm, cognitive control procedures are only modulating correct-goal fixations, and that the incorrect-goal effects are not ‘true’ effects. At first glance, this may seem feasible, given that the effects reported in Hsu and Novick also appeared more stable for correct-goal fixations than for incorrect goal-fixations. In fact, such a conclusion would seem tempting if one were to only examine monolingual performance. However, the bilinguals in our study clearly showed a modulation of the effects for incorrect-goal fixations, making this explanation unlikely. Regardless, the results in our study do suggest that cognitive control (as manifested via Stroop-related conflict) differentially affects engagement and disengagement processes associated with sentence comprehension.

Another possibility is that the monolinguals were globally more skilled, as they had overall higher correct-goal fixations and lower incorrect-goal fixations, as well as higher verbal fluency and working memory than bilinguals (see Table 1). In fact, for monolinguals, working memory resources mediated overall incorrect-goal fixations, such that those with higher working memory considered the incorrect goal less during the revision phase. In previous research, both vocabulary size and working memory have been associated with better linguistic abilities more generally [84,86,87]. In our study, such skills may have reduced monolinguals’ susceptibility to experiencing ambiguity-related conflict, thus reducing the need to engage cognitive control. This may also partially explain why in the incorrect-goal analysis the difference between ambiguous and unambiguous sentences remained regardless of prior Stroop sequence (unlike bilinguals, who only showed an ambiguity effect for sentences that were preceded by congruent Stroop sequences). In contrast to the monolingual sample in our study, the sample in Hsu and Novick [23] may have been more heterogeneous with respect to the linguistic and cognitive characteristics. However, it is not possible to directly assess this issue, as no measures of general linguistic and non-linguistic ability (beyond the main task) were reported in Hsu and Novick [23]. We therefore fully encourage future research to consider characterizing variation in language processing in order to better understand the dynamics of conflict adaptation.

While the issue of improved global skills can partly account for the effects of ambiguity and conflict adaptation that were observed in the incorrect-goal analysis, it does not explain why (and how) the incorrect-goal results yielded differences given that the same effects were similar for both groups in the correct-goal analysis. Our position is that such discrepancies are more likely to primarily reflect differences in cognitive control recruitment strategies. For monolinguals, the process of disengaging incorrect-goal interpretations following incongruent-Stroop sequences did not emerge until after the disambiguating region (i.e., “onto the box”). For bilinguals, however, this process began earlier, immediately after hearing the first prepositional phrase (i.e., “on the napkin”). This suggests that both groups relied on different linguistic cues to initiate cognitive control recruitment. Monolinguals may have used these resources more selectively and reactively, waiting for an explicit cue (i.e., the disambiguating region) that would force a revision. Bilinguals, on the other hand, may have relied more on a proactive strategy for the incorrect goal, utilizing cognitive control resources to not only recover from ambiguity, but to also identify early linguistic cues (i.e., the omission of the complementizer “that’s” during ambiguous trials) as quickly and efficiently as possible.

Another important group discrepancy in the incorrect-goal analysis was observed in how working memory modulated fixation patterns. Recall that for monolinguals, better working memory resulted in reduced incorrect-goal fixations more generally, suggesting that these resources enabled those with high working memory ability to be more efficient at disregarding incorrect-goal interpretations. This is consistent with previous psycholinguistic research suggesting that working memory measures reflect linguistic processing skills, rather than a separate domain-general construct [88,89,90]. Under this experience-based view, better working memory results from increased exposure to the distributional patterns that shape language use and comprehension, which positively affects the quality of linguistic representations, thus leading to more efficient processing.

Despite the monolingual results, the effect of working memory for bilinguals was the exact opposite: those with better working memory abilities considered incorrect-goal interpretations more, and this was especially true for ambiguous sentences. This is consistent with studies showing that individuals with greater working memory abilities become slower to process complex sentences involving ambiguity [89,91]. Following the experience-based view, it has been argued that increased linguistic experience strengthens the processing of frequently occurring linguistic patterns found in the input, which can lead to greater processing costs when dealing with less frequent patterns [92]. However, such an explanation seems unlikely to fully account for the working memory results reported here, since this would imply that the bilinguals in our study had greater cumulative linguistic experience with their L2 than the monolinguals with their L1. Despite experiencing increased English exposure due to L2 immersion, relative to monolinguals, bilinguals had overall reduced fixations on the correct goal, as well as overall increased fixations on the incorrect goal. Bilinguals also showed higher verbal fluency in their L1 relative to their L2, indicating that these individuals had, for the most part, maintained L1 dominance. All of these results suggest that bilinguals were less experienced in the L2 relative to monolinguals.

Therefore, it is difficult to attribute the group differences of working memory primarily to language experience and/or proficiency, since such an account would have predicted the same pattern of association for both monolinguals and bilinguals. Instead, the bilingual results are consistent with the idea that greater working memory resources increase susceptibility to interference stemming from incompatible interpretations [91]. This interpretation is further supported by the finding that the effect of working memory for bilinguals was primarily related to performance while listening to ambiguous instructions. Therefore, we consider the possibility that working memory measures such as the O-span reflect a combination of factors that include language-specific, as well as domain-general cognitive control resources [93]. More importantly, these results suggest that the way in which those resources are utilized will depend on experience as a proficient L2 speaker.

Unlike monolinguals, and unlike the results reported by Hsu and Novick [23], we found evidence suggesting that incongruent-Stroop sequences were negatively affecting bilingual performance while listening to unambiguous sentences. This pattern of results is consistent with previous research suggesting that bilinguals may recruit and depend on cognitive resources more globally (i.e., in contexts or in ways in which monolinguals would not recruit such resources). To illustrate, Blumenfeld and Marian [66] found that for bilinguals, and not monolinguals, the magnitude of the Stroop effect (as measured by a non-linguistic version of the Stroop task) was associated with the ability to resolve within-language interference in a separate visual-world task. In another study by Ramírez and colleagues [94], bilingual, but not monolingual, infants engaged brain regions that are associated with executive functions in order to successfully discriminate syllables from their two languages. Arguably, this is because for bilinguals the act of learning and using a language is inherently a competitive process [61]. In our study, the strategies observed in bilinguals may have further stemmed from the fact that they were performing a highly demanding task in their less experienced (although highly proficient) L2. In this sense, the group differences observed with incorrect-goal fixation patterns should not be taken as indicative of an advantage. If anything, monolinguals were more advantaged in terms of characteristics and overall performance in the main task. Instead, such differences likely reflect adaptive changes that seek to maximize the efficiency of goal-directed behavior.

More generally, the incorrect-goal results for bilinguals also revealed a dissociation in the recruitment of engagement and disengagement processes, suggesting that it was the disengagement portion that was most effortful, therefore requiring a greater need for cognitive control. This is consistent with recent studies highlighting the role of disengagement for bilinguals in linguistic and non-linguistic contexts. For example, Blanco-Elorrieta and colleagues [95] recorded brain activity of a group of bimodal bilinguals (who could sign and speak at the same time) while performing a language switching task. The results showed an engagement of (and stronger connectivity between) brain regions associated with domain-general cognitive/attentional control when switching from a ‘simultaneous’ language-context (i.e., a context where both languages were used simultaneously) to a single-language context, but not when switching contexts in the opposite direction (i.e., from a single-language context to a simultaneous context). Similar to this pattern was the result in the present study showing increased incorrect-goal fixations for bilinguals in the incongruent-unambiguous condition (where participants transitioned from a conflict-prone condition to a less demanding condition). Although one must be careful in drawing comparisons between studies with different methodologies (language vs. task switching) and between different bilingual populations (unimodal vs. bimodal bilinguals), the overall pattern of results from Blanco-Elorrieta and colleagues [95], as well as ours, seem to suggest that the process of ‘moving away’ from conflict is critical for understanding the consequences of using more than one language.

The idea that bilingualism impacts disengagement has been expressed in recent work investigating differences in attentional control between bilinguals and monolinguals. In another recent study by Grundy and Bialystok [68], monolinguals and unimodal bilinguals performed a non-linguistic switching task containing univalent sequences (where each task had its unique set of features) and bivalent sequences (where a given trial had a feature that overlapped with a previous trial from one of the other tasks). Electrophysiological results indicated that, after experiencing conflict in the bivalent sequences, bilinguals resolved the post-conflict costs more quickly and more efficiently. This finding is similar to the main results reported by Blumenfeld and Marian [66]. In this study, monolinguals and bilinguals’ eye-movements were tracked using a visual world paradigm. Participants viewed a display that contained within-language phonological competitors, which were followed by priming probe trials that measured the degree of residual phonological interference. On picture-display trials, eye-movements results showed that both groups were similarly affected by the phonological competitors. However, on subsequent priming probe trials, bilinguals’ behavioral performance was less affected by the residual interference that had emerged from the phonological competitors, suggesting that bilinguals were able to resolve prior linguistic conflict more quickly in order to engage in new task-relevant information. This is perhaps the study that aligns the closest with the results from our study, both suggesting differences in the timing of cognitive control involvement. Taken together, these studies indicate that there are circumstances in which bilinguals are able (or required) to use cognitive control procedures to disengage attention more efficiently, be it faster conflict resolution or reduced interference costs.

A final important issue to consider in the current set of results is the role of language immersion status. The bilinguals tested in the present study all came from a non-English speaking country, but became immersed in an English context later in their lives as part of their college education, while still maintaining dominance in their L1 (despite having restricted L1 exposure and use, as Table 1 suggests). A group of bilinguals with similar characteristics was examined in Zirnstein and colleagues [14], who found that, for L2 immersed bilinguals (who were also L1 dominant), the ability to generate (and recover from) prediction errors in the L2 was mediated by a combination of cognitive control and language regulatory abilities. As such, the results by Zirnstein and colleagues [14] suggested that the experiences associated with becoming immersed in an L2 environment seems to impose unique demands on the language and cognitive system. Although preliminary, the findings reported here converge with this idea, and tentatively suggest that immersion status may at least be partially responsible for the group differences in cognitive control recruitment strategies.

## 5. Conclusions

The present study provides support for the notion of adaptive changes in cognitive control as a function of language experience, but in a different form. Namely, our findings tentatively suggest that there may be a dynamic interplay between the long-term consequences of using a second language and the short-term recruitment of domain general resources that mediate processing in that same language. However, we note that the way in which this interplay plays out may vary across different bilingual populations.

It is also worth noting that the bilinguals in our study spoke different L1s, making it difficult to examine whether there were aspects of the L1 that influenced conflict adaptation in the L2. For example, it is possible that bilinguals whose languages are more likely to create conditions of cross-language conflict are also more likely to create a greater need for cognitive control recruitment. Likewise, we acknowledge the limitations that may come from comparing monolinguals and bilinguals [96], as more recent research suggests that different bilingual language experiences can come to have different consequences for both cognitive [97,98,99,100] and language [101] processes. However, it is also worth pointing out that there are situations in which such comparisons reveal important aspects of language and cognitive functioning that may be harder to interpret otherwise, at least initially.

As such, our goal in this paper was not to identify advantages or to single out individual cognitive mechanisms. Rather, our aim was to exploit how multiple cognitive processes coordinate with one another to support language processing in real time, using a different framework from what many of the previous bilingual studies have used. We believe that future research will need to adopt a similar approach in order to develop a causal theory of bilingualism and its consequences for language and cognition.

## Figures and Tables

**Table 1:** Participant characteristics.

	Monolinguals		Bilinguals	
	Valid *N*	M (SD)	Valid *N*	M (SD)
**Age, years**	25	21.40 (2.77)	24	23.96 (3.24) **
**L2 age of acquisition**	N/A	N/A	24	6.29 (3.03)
**English immersion, years**	N/A	N/A	22	1.52 (1.52)
**Self-rated exposure**				
L1	25	10.00 (0.00)	22	5.05 (2.30) ††
L2	N/A	N/A	24	8.46 (2.45)
**Self-rated use**				
L1	25	10.00 (0.00)	22	5.55 (2.41) ††
L2	N/A	N/A	24	8.17 (2.65)
**Self-rated proficiency**				
L1	25	9.66 (0.53)	22	9.87 (0.44) ††
L2	N/A	N/A	22	8.67 (0.91)
**Verbal Fluency**				
L1	23	58.22 (8.38)	21	49.86 (10.26) †
L2	N/A	N/A	24	45.04 (8.61) **
**O-span**				
recall score	26	51.04 (5.06)	23	40.35 (8.41) **
response time (ms)	26	2120.48 (297.94)	24	2155.04 (343.47)

Note: Self-ratings were made on a 10-point scale ranging from 1 (no exposure/use/proficiency) to 10 (high exposure/use/proficiency). Fluency score was measured as the average number of exemplars produced across semantic categories. O-span recall score was measured as the number of correctly recalled to-be-remembered English words out of 60 possible. O-span response time indicates the amount of time it took to correctly solve an equation. Some data were excluded due to experimental or equipment error. ** = significant differences between monolinguals and bilinguals at *p* < 0.01 level; † = marginally significant difference between bilinguals’ L1 and L2; †† = significant differences between bilinguals’ L1 and L2 at *p* < 0.01 level.

**Table 2:** Mean proportion of fixations by group, condition, and object.

	Monolinguals		Bilinguals	
**Correct Goal**	Mean	SD	Mean	SD
Congruent-Unambiguous	0.51	0.08	0.46	0.06
Incongruent-Unambiguous	0.48	0.06	0.44	0.07
Congruent-Ambiguous	0.41	0.07	0.36	0.05
Incongruent-Ambiguous	0.47	0.07	0.41	0.07
**Incorrect Goal**				
Congruent-Unambiguous	0.05	0.03	0.07	0.03
Incongruent-Unambiguous	0.07	0.03	0.10	0.04
Congruent-Ambiguous	0.13	0.06	0.17	0.07
Incongruent-Ambiguous	0.10	0.04	0.12	0.06

**Table 3:** Mean and standard deviation for Stroop performance by experimental half.

	Monolinguals		Bilinguals	
	First Half	Second Half	First Half	Second Half
**Accuracy**				
congruent	0.94 (0.07)	0.92 (0.12)	0.86 (0.09)	0.85 (0.11)
incongruent	0.90 (0.07)	0.94 (0.06)	0.80 (0.10)	0.86 (0.11)
**Response Time (ms)**				
congruent	657.50 (58.58)	713.75 (66.03)	645.60 (55.56)	686.93 (57.49)
incongruent	684.68 (72.19)	680.44 (70.03)	664.38 (72.19)	648.28 (58.68)

## Supplementary Materials

The following are available online at https://www.mdpi.com/2076-3425/9/5/95/s1, Figure S1: Mean proportion of fixations on the correct goal over time as a function of ambiguity for monolinguals (top) and bilinguals (bottom). Higher values on the y-axis indicate more looks to the correct goal. Colored error bars indicate 95% confidence intervals, Figure S2: Mean proportion of fixations on the correct goal over time as a function of ambiguity and preceding Stroop trial type for monolinguals (top row) and bilinguals (bottom row). Colored error bars indicate 95% confidence intervals, Figure S3: Mean proportion of fixations on the incorrect goal over time as a function of ambiguity. Higher values on the y-axis indicate more looks to the incorrect goal. Colored error bars indicate 95% confidence intervals, Figure S4: Mean proportion of fixations on the incorrect goal over time as a function of ambiguity and preceding Stroop trial type. Colored error bars indicate 95% confidence intervals, Table S1: Summary of mixed model analysis for correct goal fixations, Table S2: Summary of mixed model analysis for incorrect goal fixations, Table S3: Summary of mixed model analysis for correct goal fixations by experimental half, Table S4: Summary of mixed model analysis for incorrect goal fixations by experimental half, Table S5: Summary of mixed model analysis examining the effect of working memory on incorrect goal fixations. The stimuli materials have been made publicly available by Hsu and Novick [23].
