# Supplementary material for: Cognitive Control Facilitates Attentional Disengagement during Second Language Comprehension
Source: Brain Sci. 2019 Apr 27;9(5):95. doi: 10.3390/brainsci9050095 (PMC6562798; doi:10.3390/brainsci9050095)
Supplement: Supplementary file 1 [file brainsci-09-00095-s001.pdf]

Supplementary Materials: Cognitive Control Facilitates Attentional Disengagement during Second Language Comprehension

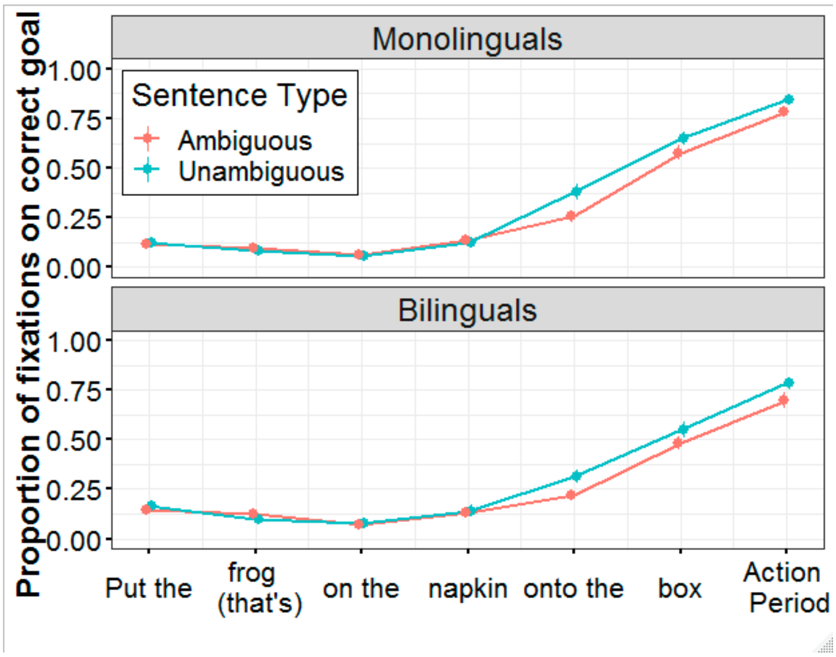

Figure S1. Mean proportion of fixations on the correct goal over time as a function of ambiguity for monolinguals (top) and bilinguals (bottom). Higher values on the y-axis indicate more looks to the correct goal. Colored error bars indicate 95% confidence intervals.

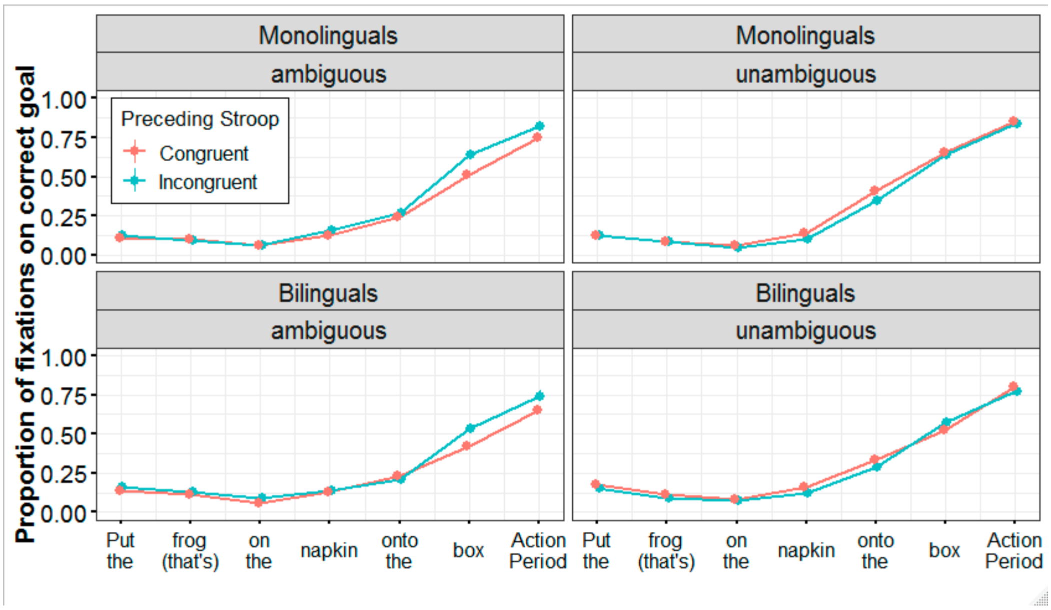

Figure S2. Mean proportion of fixations on the correct goal over time as a function of ambiguity and preceding Stroop trial type for monolinguals (top row) and bilinguals (bottom row). Colored error bars indicate 95% confidence intervals.

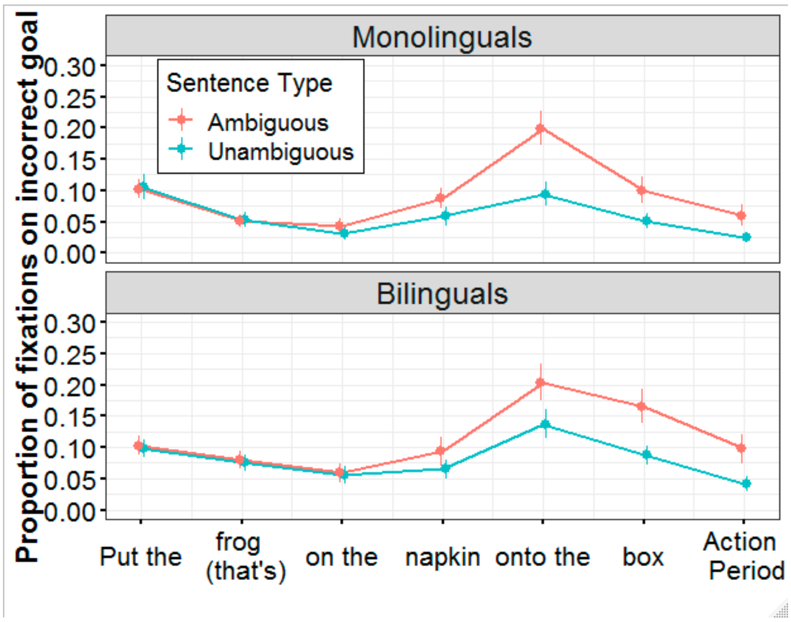

Figure S3. Mean proportion of fixations on the incorrect goal over time as a function of ambiguity. Higher values on the y-axis indicate more looks to the incorrect goal. Colored error bars indicate 95% confidence intervals.

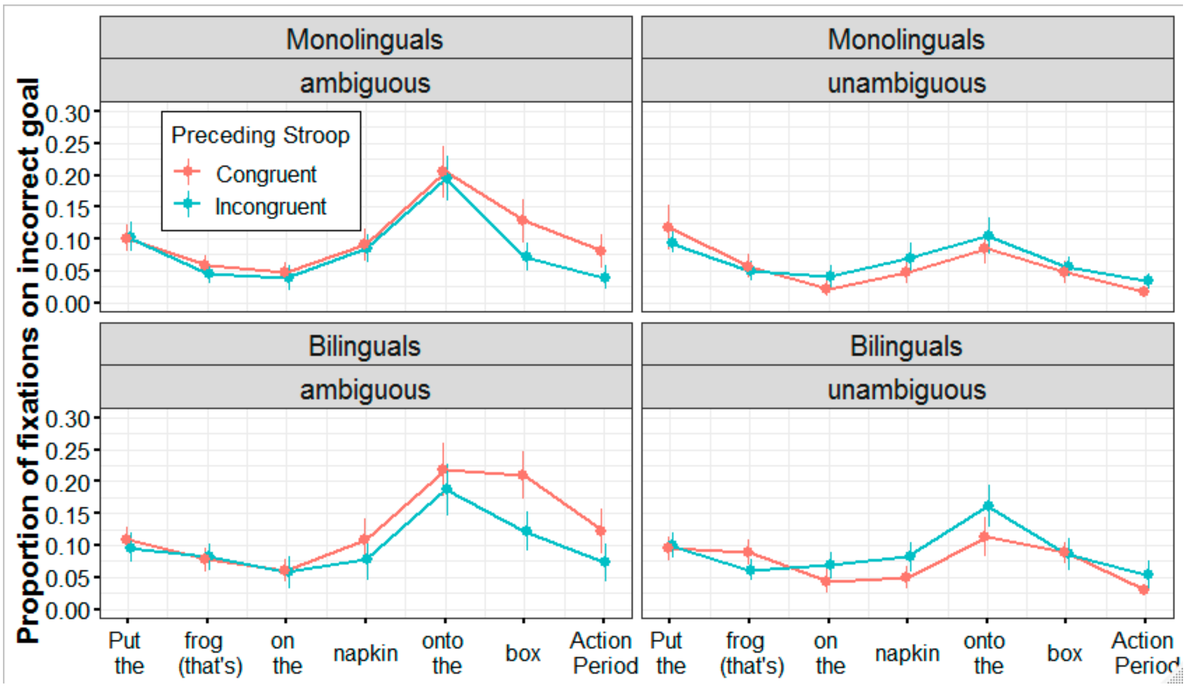

Figure S4. Mean proportion of fixations on the incorrect goal over time as a function of ambiguity and preceding Stroop trial type. Colored error bars indicate 95% confidence intervals.

Table S1. Summary of mixed model analysis for correct goal fixations.

Formula: Elog(PropLooks) ~ Stroop × Ambiguity × Group + (Stroop × Ambiguity ∣ participant) + (Stroop × Ambiguity ∣ item)				
**Fixed effects**	**Estimate**	***SE***	***t*-values**	***p***
(Intercept)	−0.11	0.02	-6.29	<0.001
Stroop	0.03	0.02	1.52	0.137
**Ambiguity**	**0.09**	**0.01**	**6.05**	**<0.001**
**Group**	**0.09**	**0.03**	**3.43**	**0.001**
**Stroop:Ambiguity**	**−0.10**	**0.03**	**−3.02**	**0.004**
Stroop:Group	0.01	0.02	0.32	0.747
Ambiguity:Group	−0.01	0.02	-0.62	0.539
Stroop:Ambiguity:Group	−0.05	0.04	-1.26	0.212
**Random effects**	**Variance**	***SD***		
Intercept ∣ participant	0.0070	0.08		
Stroop ∣ participant	0.0004	0.02		
Ambiguity ∣ participant	0.0011	0.03		
Stroop:Ambiguity ∣ participant	0.0017	0.04		
Intercept ∣ item	0.0064	0.08		
Stroop ∣ item	0.0083	0.09		
Ambiguity ∣ item	0.0013	0.04		
Stroop:Ambiguity ∣ item	0.0084	0.09		
Residual	0.0592	0.24		

Note: SE = standard error of coefficient estimate. Contrast coding was used for all factors, making the coefficient interpretations as follows: intercept = estimated overall mean fixations on the correct goal (in logits); Stroop = mean difference between trials that were preceded by congruent or by incongruent Stroop sequences; Ambiguity = mean difference between ambiguous and unambiguous sentences; Group = mean difference between bilinguals and monolinguals.

Table S2. Summary of mixed model analysis for incorrect goal fixations.

Formula: Elog(PropLooks) ~ Stroop × Ambiguity × Group + (Stroop+Ambiguity ∣ participant) + (Stroop × Ambiguity + Group ∣ item)				
**Fixed effects**	**Estimate**	***SE***	***t*-values**	***p***
(Intercept)	−0.84	0.02	−38.20	< 0.001
Stroop	−0.03	0.02	-1.35	0.184
**Ambiguity**	**−0.13**	**0.02**	**−5.61**	**<0.001**
**Group**	**−0.07**	**0.02**	**−2.95**	**0.005**
**Stroop:Ambiguity**	**0.09**	**0.04**	**2.07**	**0.046**
Stroop:Group	0.02	0.02	0.72	0.471
Ambiguity:Group	0.00	0.03	0.12	0.907
**Stroop:Ambiguity:Group**	**−0.08**	**0.04**	**−1.92**	**0.055**
**Random effects**	**Variance**	***SD***		
Intercept ∣ participant	0.0056	0.07		
Stroop ∣ participant	0.0000	0.01		
Ambiguity ∣ participant	0.0052	0.07		

Intercept ǀ item	0.0153	0.12
Stroop ǀ item	0.0068	0.08
Ambiguity ǀ item	0.0108	0.10
Stroop:Ambiguity ǀ item	0.0003	0.02
Group ǀ item	0.0136	0.12
Residual	0.0631	0.25

Note: Contrast coding was used for all factors, making the coefficient interpretations as follows: intercept = estimated overall mean fixations on the incorrect goal (in logits); Stroop = mean difference between trials that were preceded by congruent or by incongruent Stroop sequences; Ambiguity = mean difference between ambiguous and unambiguous sentences; Group = mean difference between bilinguals and monolinguals.

Table S3. Summary of mixed model analysis for correct goal fixations by experimental half.

Formula: Elog(PropLooks) ~ Stroop × Ambiguity × Group × Experimental Half + (1ǀ participant:Stroop:Ambiguity) + (1 ǀ item:Stroop:Ambiguity:Group)				
**Fixed effects**	**Estimate**	***SE***	***t*-values**	***p***
(Intercept)	−0.16	0.01	−12.42	<0.001
Stroop	0.04	0.03	1.69	0.092
Ambiguity	0.14	0.03	5.28	0.000
Group	0.08	0.03	3.18	0.002
EH2	0.11	0.02	6.65	0.000
**Stroop:Ambiguity**	**−0.18**	**0.05**	**−3.38**	**0.001**
Stroop:Group	0.04	0.05	0.74	0.458
Ambiguity:Group	0.00	0.05	−0.08	0.939
Stroop:EH2	−0.08	0.03	−2.35	0.020
Ambiguity:EH2	−0.09	0.03	−2.68	0.008
Group:EH2	0.01	0.03	0.40	0.690
Stroop:Ambiguity:Group	0.04	0.10	0.34	0.731
**Stroop:Ambiguity:EH2**	**0.17**	**0.06**	**2.66**	**0.009**
Stroop:Group:EH2	−0.07	0.06	−1.09	0.278
Ambiguity:Group:EH2	−0.02	0.06	−0.28	0.783
Stroop:Ambiguity:Group:EH2	−0.16	0.13	−1.21	0.228
**Random effects**	**Variance**	***SD***		
Intercept ǀ participant:Stroop:Ambiguity	0.0075	0.09		
Intercept ǀ item:Stroop:Ambiguity:Group	0.0071	0.08		
Residual	0.0583	0.24		

Note: EH2 = second experimental half. First experimental half was set as the reference level, yielding the following interpretation for the intercept = estimated overall mean fixations on the correct goal (in logits) during the first experimental half.

Table S4. Summary of mixed model analysis for incorrect goal fixations by experimental half.

Formula: Elog(PropLooks) ~ Stroop × Ambiguity × Group × Experimental Half + (Stroop + Ambiguity ∣ participant) + (Stroop × Ambiguity + Group ∣ item)				
**Fixed effects**	**Estimate**	***SE***	***t*-values**	***p***
(Intercept)	−0.84	0.01	−66.25	<0.001
Stroop	0.01	0.03	0.37	0.712
Ambiguity	−0.11	0.03	−4.50	<0.001
Group	−0.07	0.03	−2.77	0.006
EH	0.08	0.02	3.74	<0.001
Stroop:Ambiguity	0.09	0.05	1.81	0.072
Stroop:Group	0.03	0.05	0.50	0.615
Ambiguity:Group	0.00	0.05	0.08	0.934
Stroop:EH	−0.03	0.05	−0.69	0.494
Ambiguity:EH	−0.10	0.05	−2.27	0.024
Group:EH	0.00	0.05	−0.01	0.989
**Stroop:Ambiguity:Group**	**−0.09**	**0.05**	**−2.07**	**0.038**
**Stroop:Ambiguity:EH**	**0.23**	**0.09**	**2.59**	**0.010**
Stroop:Group:EH	−0.02	0.09	−0.18	0.857
Ambiguity:Group:EH	−0.07	0.09	−0.79	0.430
Stroop:Ambiguity:Group:EH	−0.04	0.18	−0.21	0.831
**Random effects**	**Variance**	***SD***		
Intercept ∣ participant:Stroop:Ambiguity	0.0067	0.08		
Intercept ∣ item:Stroop:Ambiguity:Group	0.0179	0.13		
Residual	0.0641	0.25		

Note: EH = experimental half. All factors were contrast coded, yielding the following interpretation for the intercept = estimated overall mean fixations on the incorrect goal (in logits).

Table S5. Summary of mixed model analysis examining the effect of working memory on incorrect goal fixations.

Formula: Elog(PropLooks) ~ Stroop × Ambiguity × Group × Experimental Half × Working Memory + (1 ∣ participant:Stroop:Ambiguity) + (1 ∣ item:Stroop:Ambiguity:Group)				
**Fixed effects**	**Estimate**	***SE***	***t*-values**	***p***
(Intercept)	−0.82	0.01	−56.87	<0.001
Stroop	0.02	0.03	0.73	0.465
Ambiguity	−0.13	0.03	−4.65	<0.001
Group	−0.05	0.03	−1.76	0.080
EH	0.12	0.02	4.82	<0.001
WM	−0.02	0.01	−1.55	0.124
Stroop:Ambiguity	0.10	0.06	1.68	0.094
Stroop:Group	0.03	0.06	0.59	0.554
Ambiguity:Group	−0.00	0.06	−0.05	0.957
Stroop:EH	−0.07	0.05	−1.35	0.179
Ambiguity:EH	−0.15	0.05	−2.96	0.003
Group:EH	0.02	0.05	0.42	0.672

Stroop:WM	−0.01	0.02	−0.33	0.744
Ambiguity:WM	0.01	0.02	0.31	0.756
**Group:WM**	**−0.09**	**0.02**	**−3.60**	**<0.001**
EH:WM	−0.02	0.02	−1.21	0.225
Stroop:Ambiguity:Group	−0.05	0.11	−0.47	0.638
Stroop:Ambiguity:EH	0.30	0.10	2.97	0.003
Stroop:Group:EH	−0.08	0.10	−0.83	0.405
Ambiguity:Group:EH	−0.09	0.10	−0.91	0.361
Stroop:Ambiguity:WM	−0.02	0.05	−0.49	0.627
Stroop:Group:WM	−0.04	0.05	−0.83	0.409
Ambiguity:Group:WM	0.06	0.05	1.35	0.180
Stroop:Block:WM	0.06	0.03	1.70	0.089
Ambiguity:Block:WM	0.02	0.03	0.56	0.573
**Group:Block:WM**	**−0.12**	**0.03**	**−3.49**	**<0.001**
Stroop:Ambiguity:Group:EH	0.04	0.20	0.18	0.859
Stroop:Ambiguity:Group:WM	−0.02	0.09	−0.18	0.858
Stroop:Ambiguity:EH:WM	−0.07	0.07	−0.97	0.330
Stroop:Group:Block:WM	0.12	0.07	1.82	0.069
**Ambiguity:Group:EH:WM**	**0.15**	**0.07**	**2.19**	**0.029**
Stroop:Ambiguity:Group:EH:WM	−0.21	0.14	−1.51	0.130
**Random effects**	**Variance**	***SD***		
Intercept | participant:Stroop:Ambiguity	0.0055	0.07		
Intercept | item:Stroop:Ambiguity:Group	0.0183	0.14		
Residual	0.0634	0.25		

Note: EH = experimental half; WM = O-span recall score. WM was centered using a *z*-score transformation. All other factors were contrast coded, yielding the following interpretation for the intercept = estimated overall mean fixations on the incorrect goal (in logits) for individuals with average working memory.
